# Network meta-analysis made simple: A composite likelihood approach

**DOI:** 10.1017/rsm.2024.12

**Published:** 2025-03-17

**Authors:** Yu-Lun Liu, Bingyu Zhang, Haitao Chu, Yong Chen

**Affiliations:** 1 Peter O’Donnell Jr. School of Public Health, University of Texas Southwestern Medical Center, Dallas, TX, USA; 2 Center for Health AI and Synthesis of Evidence, Perelman School of Medicine, University of Pennsylvania, Philadelphia, PA, USA; 3 Applied Mathematics and Computational Science, University of Pennsylvania, Philadelphia, PA, USA; 4 Statistical Research and Data Science, Pfizer Inc., New York, NY, USA; 5 Division of Biostatistics, University of Minnesota Twin Cities, Minneapolis, MN, USA; 6 Leonard Davis Institute of Health Economics, Philadelphia, PA, USA; 7 Penn Medicine Center for Evidence-based Practice, Philadelphia, PA, USA; 8 Penn Institute for Biomedical Informatics, Philadelphia, PA, USA

**Keywords:** composite likelihood, indirect evidence, meta-analysis, network meta-analysis, unknown within-study correlations

## Abstract

Network meta-analysis (NMA), also known as mixed treatment comparison meta-analysis or multiple treatments meta-analysis, extends conventional pairwise meta-analysis by simultaneously synthesizing multiple interventions in a single integrated analysis. Despite the growing popularity of NMA within comparative effectiveness research, it comes with potential challenges. For example, within-study correlations among treatment comparisons are rarely reported in the published literature. Yet, these correlations are pivotal for valid statistical inference. As demonstrated in earlier studies, ignoring these correlations can inflate mean squared errors of the resulting point estimates and lead to inaccurate standard error estimates. This article introduces a composite likelihood-based approach that ensures accurate statistical inference without requiring knowledge of the within-study correlations. The proposed method is computationally robust and efficient, with substantially reduced computational time compared to the state-of-the-science methods implemented in R packages. The proposed method was evaluated through extensive simulations and applied to two important applications including an NMA comparing interventions for primary open-angle glaucoma, and another comparing treatments for chronic prostatitis and chronic pelvic pain syndrome.

## Highlights

**What is already known?**

- Network meta-analysis extends conventional pairwise meta-analysis by simultaneously synthesizing multiple interventions in a single integrated analysis.
- A significant challenge in network meta-analysis is the lack of reported within-study correlations among treatment comparisons in published studies.

**What is new?**

- We propose a new method for network meta-analysis that ensures valid statistical inference without the need for knowledge of within-study correlations.
- The proposed method employs a composite likelihood and a sandwich-type robust variance estimator, offering a computationally efficient and scalable solution, particularly for network meta-analysis involving a large number of treatments and studies.

**Potential impact for *Research Synthesis Methods* readers**

- The proposed method can be easily applied to any univariate network meta-analysis project without requiring knowledge of within-study correlations among treatment comparisons.

## 1 Introduction

Meta-analysis is a widely used tool in systematic reviews for combining and contrasting multiple studies to obtain overall estimates of the relative effects in the target population.^1^ ^,^ ^2^ The methodology of pairwise meta-analysis, which focuses solely on comparing an intervention with a reference (e.g., control or placebo), has been well developed.^3^ ^,^ ^4^ For many medical conditions, there are often more than two interventions of interest. In such situations, performing isolated pairwise meta-analysis might neither adequately represent the comprehensive landscape of interventions nor guide the selection of optimal treatment to maximize patient benefits.^5^ ^,^ ^6^ Furthermore, it is often unrealistic to anticipate that there will be at least a head-to-head trial comparing any two interventions of interest.

Network meta-analysis (NMA), coined by Lumley back in 2002,^7^ also known as multiple treatment meta-analysis or mixed treatment comparison, is a state-of-the-science technique for making inferences about multiple treatments. This approach enables the comparison of diverse treatment subsets across various trials. A notable application of NMA is the comparison of treatment options for depression conducted by Cipriani and his colleagues,^8^ which provided a comprehensive summary of the relative efficacy and safety of 21 antidepressant drugs based on all available studies to date. Their insights have the potential to influence clinical practices, impacting millions of individuals who suffer from depression globally. Essentially, the rationale of NMA is to expand pairwise meta-analysis to simultaneously compare multiple treatments and produce consistent estimates of relative treatment effects by synthesizing both direct and indirect clinical evidence in a single integrated analysis.^6^

Over the last two decades, a plethora of methodological developments in NMA has emerged, mostly focusing on meta-regression and Bayesian hierarchical models, among others.^9^ ^–^ ^12^ For instance, the estimation of indirect evidence in NMAs was first derived through a meta-regression model,^7^ ^,^ ^13^ wherein various treatment comparisons were treated as covariates in a model. Yet, such an approach can be challenging to estimate between-study variance, especially for sparse networks, so that the between-study heterogeneity variance is often assumed to be common across all treatment comparisons in a network.^7^ ^,^ ^13^ ^,^ ^14^ On the other hand, the assumption of common between-study heterogeneity variance can lead to inaccurate estimates.^15^ A shift toward the Bayesian hierarchical model, pioneered by Lu and his colleagues,^16^ ^,^ ^17^ has received significant interest. Their subsequent work^18^ further discussed how the consistency equations imposed restrictions on between-study heterogeneity of each treatment comparison and used the spherical parameterization based on Cholesky decomposition to implement the constraints.

In addition to the contrast-based NMA (CB-NMA), which focuses on the (weighted) average of study-specific relative effects by assuming fixed study-specific intercepts, arm-based NMA (AB-NMA) models assess study-specific absolute effects and assume random intercepts. This offers greater flexibility in estimands, including both population-averaged absolute and relative effects.^19^ ^–^ ^23^ Alternatively, NMAs can be conceptualized as a multivariate meta-analysis.^24^ Other existing models for implementing NMAs include electrical networks and graph-theoretical methods^25^ ^,^ ^26^ under fixed-effects or random-effects models. In addition to the classical framework of synthesizing point estimates, a novel confidence distribution framework, based on a sample-dependent distribution function, has been proposed.^27^

Despite the popularity of NMAs, an important challenge has not been fully addressed: the unknown or unreported within-study correlations. Specifically, estimates of contrasts between each pair of treatment comparisons are correlated within a multi-arm study when these contrasts involve a common comparator. On the other hand, within-study correlations are rarely reported in published trials.^28^ When conducting an NMA, ignoring within-study correlations can lead to biased estimates of relative treatment effects; particularly, these estimates were also found to have increased mean-square errors and standard errors.^28^ ^,^ ^29^

When the impact of within-study correlations is non-ignorable, several methods have been proposed and used to obtain estimates of within-study correlations. First, the availability of individual participant-level data allows to compute the within-study correlations directly between treatment comparisons in each trial;^30^ however, it is uncommon in meta-analysis to have individual participant-level data for all trials. Study investigators are often unable to provide information about within-study correlations even if we make requests directly.^31^ Second, an alternative method, known as the Pearson correlation method, proposed by Kirkham et al.,^32^ can be implemented in multivariate meta-analysis. Third, Riley et al.^33^ proposed a single correlation parameter to capture both within-study and between-study correlations in the setting of multivariate meta-analysis. As pointed out by Riley et al.,^28^ the impact of within-study correlations is relative to the magnitude of between-study variation. In other words, when total variation in estimated effect sizes across studies, as the sum of within-study and between-study covariance, is dominated by within-study variation, the impact of within-study correlations can be substantial. Ignoring the unknown within-study correlations can lead to misleading results.^33^ Other methods, such as Bayesian approaches,^34^ have also been proposed.

Even though the within-study correlations can be estimated by the abovementioned methods, each of them requires additional assumptions and constraints, along with high computational complexity, to ensure that the estimated within-study variance–covariance matrices are valid and positive definite, particularly for Bayesian methods using Markov chain Monte Carlo algorithms. One of the existing methods to resolve such an issue is to restrict the range of correlation coefficients in each study from a truncated prior distribution so that the positive definiteness of the variance–covariance matrix is guaranteed.^35^ Other alternatives, such as Cholesky parameterization and spherical decomposition,^36^ have been employed to ensure positive-definite variance–covariance matrices for meta-analysis and NMA under a Bayesian framework. These methods, however, may be more difficult to implement in NMAs as the number of studies and treatments in a network grows.

To overcome the aforementioned challenges, we propose a new method without imposing any additional assumptions beyond those in a standard NMA. Compared to the conventional approach implementing NMAs with the standard full likelihood, our proposed method does not require knowledge of the typically unreported within-study correlations among treatment comparisons. Using composite likelihood^37^ ^,^ ^38^ and the finite-sample corrected variance estimator,^39^ ^,^ ^40^ our proposed method can lead to valid effect size estimates with coverage probabilities close to the nominal level. We also derived the corresponding algorithm which is computationally efficient and scalable to a large number of treatments in NMAs. Unlike the state-of-the-art methods, whose computational time increases exponentially with respect to the number of treatments and studies, the computational time of our algorithm increases linearly with the number of treatments and remains nearly invariant with respect to the number of studies. Further, our algorithm avoids the issue of singular covariance estimates, which is a known practical issue when conducting multivariate meta-analysis or NMA.^29^ ^,^ ^31^ ^,^ ^41^ ^–^ ^44^

The rest of the article is organized as follows. In Section 2, we give an overview of the two motivating examples, namely, an NMA comparing interventions for primary open-angle glaucoma, and an NMA comparing treatments for chronic prostatitis and chronic pelvic pain syndrome. In Section 3, we formulate the proposed method and introduce a treatment ranking procedure, while in Section 4, we describe a series of simulation studies to illustrate the limitations of conventional NMA models, and to examine the statistical properties of the proposed method. In Section 5, we present the applications of the proposed method to the two motivating examples. We conclude with a discussion and key messages in Section 6.

## 2 Two motivating examples

### 2.1 Comparison of interventions for primary open-angle glaucoma

Li et al.^45^ and Wang et al.^46^ conducted an NMA to compare all first-line treatments for primary open-angle glaucoma or ocular hypertension. Glaucoma is a disease of the optic nerve characterized by optic nerve head changes and associated visual field defects.^47^ This NMA consisted of 125 trials comparing 14 active drugs and a placebo in subjects with primary open-angle glaucoma or ocular hypertension. The studies were collected from 1983 to 2016 through the Cochrane Register of Controlled Trials, Drugs@FDA, and ClinicalTrials.gov;^46^ a total of 22,656 participants were included in these studies.

**Figure 1:**
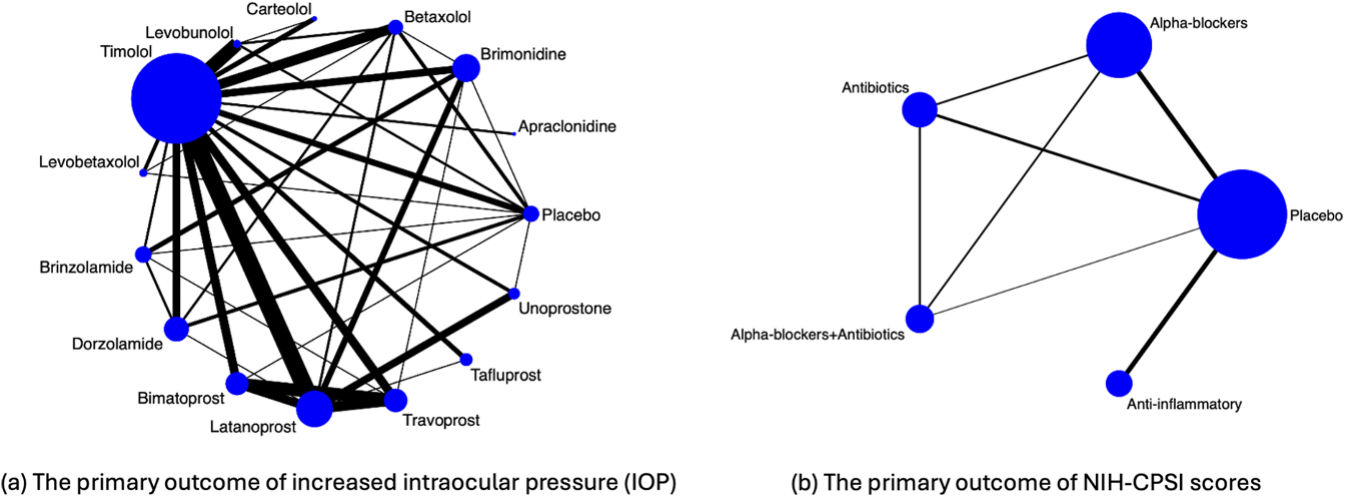
Illustration of evidence network diagrams. The size of each node is proportional to the number of participants assigned to each treatment. Solid lines represent direct comparisons between treatments in trials, with line thickness proportional to the number of trials directly comparing each pair of treatments.

Figure 1(a) visualizes the data structure. Specifically, the 14 active drugs were divided into four major drug classes, including 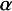 -2 adrenergic agonists, 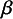 -blockers, carbonic anhydrase inhibitors, and prostaglandin analogs. This network consisted of 114 two-arm studies, 10 three-arm studies, and 1 four-arm study. The primary outcome of interest was the difference in mean increased intraocular pressure (IOP) measured by any method at 3 months in continuous millimeters of mercury (mmHg). The original NMA analysis^45^ employed a Bayesian hierarchical model with the Markov chain Monte Carlo technique.^16^ ^,^ ^17^ Their analysis focused on modeling the between-study variance–covariance matrix, assuming either a homogeneous or heterogeneous structure, rather than the within-study variance–covariance matrix. A ranking of treatments was produced through the surface under the cumulative ranking curve.^48^ The study found that all active drugs were clinically effective in reducing IOP at 3 months compared with placebo, with bimatoprost, latanoprost, and travoprost ranked as the first, second, and third most efficacious drugs, respectively, in lowering IOP.

As described in the Introduction section, an important limitation of this motivating example is the unknown or unreported within-study correlations. Specifically, contrast treatment estimates for the IOP outcome may be potentially correlated within a trial; for example, in five studies, both bimatoprost and travoprost were compared against latanoprost within the same trial.^49^ ^–^ ^53^ These within-study correlations among treatment comparisons were not reported, and individual participant-level data were unavailable for computing such correlations.

### 2.2 Comparison of treatments for the chronic prostatitis and chronic pelvic pain syndrome

Thakkinstian et al.^54^ performed an NMA to determine the effectiveness of multiple pharmacological therapies in improving chronic prostatitis symptoms in patients with chronic prostatitis and chronic pelvic pain syndrome (CP/CPPS). CP/CPPS is a common disorder characterized by two major clinical manifestations: pelvic pain and lower urinary tract symptoms.^55^ The identified studies were collected from the MEDLINE and EMBASE databases up to January 13, 2011, and included a total of 1,669 participants.

Figure 1(b) visualizes the data structure. The primary outcome of interest was the symptom score measured by the National Institutes of Health Chronic Prostatitis Symptom Index (NIH-CPSI), which consists of total symptoms, pain, voiding, and quality of life scores.^56^ The dataset consisted of 19 published trials comparing five treatment regimens, including: placebo, any 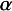 -blockers (terazosin, doxazosin, tamsulosin, alfuzosin, silodosin), any antibiotics (ciprofloxacin, levofloxacin, tetracycline), anti-inflammatory/immune modulatory agents (steroidal and non-steroidal anti-inflammatory drugs, glycosaminoglycans, phytotherapy, and tanezumab), and a combination of antibiotics and 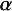 -blockers. Treatment comparisons among the five treatments were conducted by Thakkinstian et al.^54^ using an NMA approach. Their findings suggested that 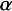 -blockers, antibiotics, and/or anti-inflammatory/immune modulatory agents were more efficacious in improving the total NIH-CPSI symptom scores compared to placebo. In this example, within-study correlations among treatment comparisons were not available for any of the included studies.

## 3 Methods

In this section, we introduce the proposed composite likelihood-based method for conducting NMA without the need for knowledge of within-study correlations. Throughout this article, we focus on the contrast-based model,^13^ although our method can be extended to arm-based models.^11^ ^,^ ^21^ ^–^ ^23^ The contrast-based model employs a two-stage estimation approach: in the first stage, the estimated effects comparing all possible intervention options in studies are computed, along with their associated standard errors from the contrast-level data, and in the second stage, the effect estimates are analyzed using a normal likelihood approximation.

### 3.1 Notations and model specification

Suppose that a network consists of *m* studies ( 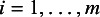 ) comparing a set of treatment options 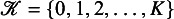 for ( 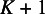 ) treatments. Each design ( 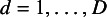 ) corresponds to a subset of treatments 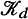 , i.e., 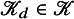 , and let 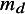 be the number of studies in design *d*. Under the assumptions of consistency, a network with ( 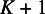 ) treatments contains *K* basic parameters. These parameters are frequently taken to be the relative effects of each treatment versus a reference (or common comparator). In this article, we do not assume that all studies utilize the same reference treatment. Instead, our proposed method incorporates all observed treatment comparisons, ensuring that reported treatment effects and standard errors contribute to the estimation of objective parameters in the proposed composite likelihood function. Moreover, the estimation does not depend on the choice of a common reference.

Suppose 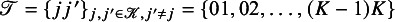 is the set of *N* observed treatment comparisons. Let 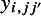 be an observed treatment effect comparing treatment *j* to treatment 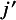 in the i-th study. Let 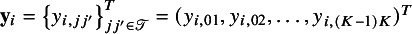 be the vector of observed contrasts of treatments, along with the vector of associated standard errors 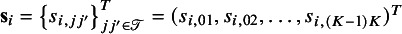 . The observed relative treatment effects in an NMA are modeled via a random-effects framework, 
(1)

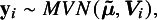




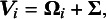




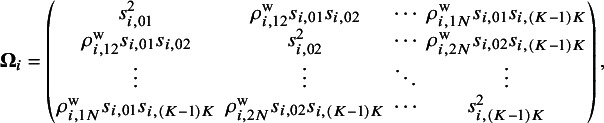




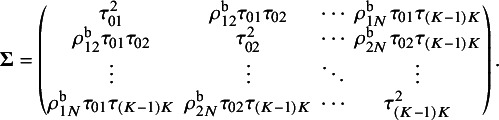

Here, 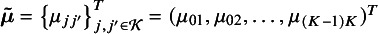 represents a vector of true population relative treatment effect sizes. The 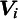 matrix indicates that the total variability affecting summary measures in each study is the sum of both within-study and between-study variance–covariance matrices. For the within-study variance–covariance 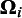, 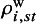 (for 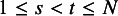 ) refers to the within-study correlation, which is rarely reported in published literature or even calculated in individual studies. We assume that 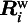 is the within-study correlation matrix, where the diagonal elements are equal to 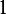 and the off-diagonal elements are 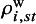 for 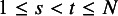 . For the between-study variance–covariance matrix 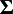, 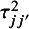 represents the heterogeneity variance of the outcome comparing treatment *j* and treatment 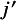 , and 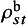 denotes the between-study correlation for 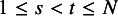 . We assume that 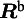 is the between-study correlation matrix, where the diagonal elements are equal to 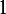 and the off-diagonal elements are 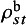 for 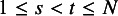 .

### 3.2 Proposed method

Let 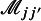 be the subset of studies that report effect sizes and standard errors for the outcome in the treatment comparison between *j* and 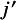 . Let 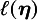 be the log composite likelihood function of the model defined in Equation (1), given the observed data 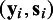 . We have 
(2)

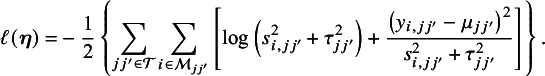



In order to fit an NMA, it is indispensable that the consistency equation is satisfied as follows, 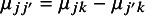 , 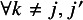 . Throughout this section, we choose treatment ‘0’ as a common reference. Then, in Equation (2), we only need to estimate the parameters 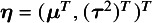, where 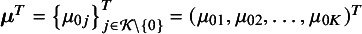 and 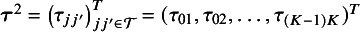. We obtain the estimates of these parameters by maximizing the log composite likelihood function, 

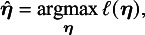

where the estimator 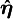 is asymptotically normal as 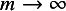 . The asymptotic variance–covariance matrix of 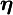 can be estimated by a sandwich-type estimator of form 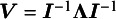, with 
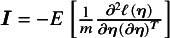
 and 
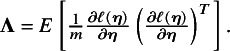
 Here, we opted for a sandwich-type variance estimator, which offers two key advantages in NMAs. First, it is robust to dependence. Even though the composite likelihood method assumes independence between treatment effects within a study, the sandwich estimator can partially account for this dependence. It achieves this by incorporating additional information during variance–covariance estimation, leading to more accurate standard errors for treatment effects. Second, the sandwich-type estimator is generally robust to misspecification of covariance structures. This robustness is particularly beneficial for handling complex data structures often encountered in NMAs. However, it is important to note that the underlying marginal model itself cannot be misspecified. We also note that the restricted maximum likelihood (REML) estimator produces the same asymptotic distribution as the maximum likelihood estimator by incorporating the extra term of 

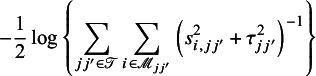

in Equation (2). Additionally, we notice that 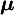 is information-orthogonal to 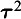. Assuming the information matrix is 
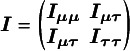
 with 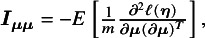 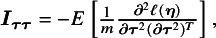 and 
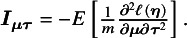
 The off-diagonal element of the information matrix, 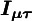, satisfies 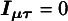, implying that 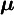 and 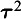 are information-orthogonal. Under this information-orthogonality property, the variance–covariance matrix of 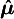 involves the information of 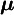 alone and can be simplified as 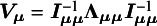, with 
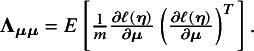
 The asymptotic variance–covariance matrix is estimated by its empirical variance–covariance matrix 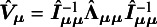, where 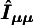 and 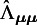 are the submatrices of 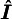 and 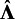, respectively. As elucidated by Liang and Zeger,^57^ the information-orthogonality property implies that the between-study variance estimates have a limited impact on the estimation of effect sizes 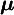. A detailed description of robust sandwich-type variance estimation is provided in Appendices 1 and 2 of the Supplementary Material.

The efficient and iterative algorithm for parameter estimation can be implemented by maximizing the log composite likelihood function in Equation (2), as described in Algorithm 1. More specifically, when 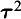 is fixed at some value of 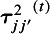, the parameters 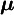 can be estimated by solving a system of linear equations. In other words, maximizing 
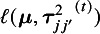
 over 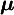 yields 

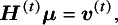

where 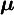 is the solution of the above system of linear equations, and 

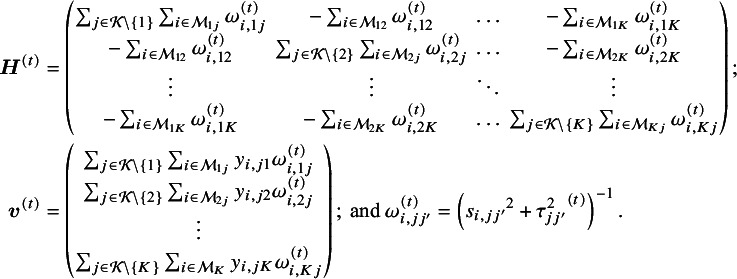



The proof of convergence for Algorithm 1 is provided in Appendix 3 of the Supplementary Material. Additionally, a detailed description of testing for inconsistency can be found in Appendix 4 of the Supplementary Material. The R code for the 3-arm study is publicly available on GitHub: https://github.com/Penncil/xmeta/tree/master/R/CLNMA.equal.tau.R.

**Figure:**
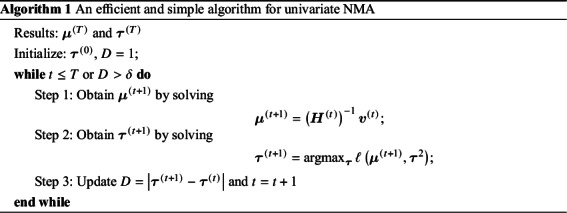

### 3.3 Treatment ranking

The hierarchy of comparable interventions can be computed by incorporating the NMA estimates obtained from the proposed composite likelihood-based method. Among various approaches to treatment ranking, the most commonly employed method relies on ranking probabilities; the probabilities for each treatment can be placed at a specific ranking position, e.g., best, second best, third best treatment, and so forth, in comparison to all other treatments in a network. These approaches for treatment ranking include the surface under the cumulative ranking curve (SUCRA)^48^ and P-score techniques,^58^ among others. In this article, we adopted the SUCRA method.^48^ By incorporating the NMA estimates obtained from our proposed method into SUCRA, we can properly account for uncertainty in the estimates of relative treatment ranking. Specifically, for each treatment *j* out of the ( 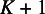 ) competing treatments, SUCRA is calculated as follows: 

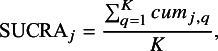

where 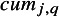 refers to the cumulative probability of being among the *q*-best treatments 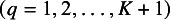 . A SUCRA value of 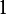 indicates that the treatment is ranked as the best, while a value of 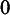 indicates the treatment is ranked as the worst.

## 4 Simulation study

In this section, our objective is to assess the impact of within-study correlations on pooled estimates when employing the proposed composite likelihood-based method. We conducted extensive simulation studies to evaluate the performance of the proposed method across various scenarios, varying factors such as the within-study correlations, between-study heterogeneity variance, and the number of studies. Furthermore, we compared the computational time of the proposed method with existing NMA methods implemented in the R packages.

### 4.1 Data-generating mechanisms

For the simulation study, we considered a contrast-based NMA consisting of a three-arm design (i.e., *A*, *B*, and *C*, where *A* is treated as the reference) for a single continuous outcome of primary interest. The simulated data were generated using the following model: 
(3)

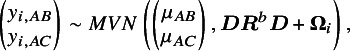

where 
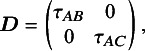
 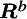 represents the 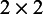 between-study correlation matrix, and 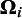 denotes the 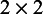 within-study variance–covariance matrix. Within the simulated network, we assumed the consistency equation, in terms of 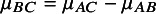 . As suggested by Lu and Ades,^17^ the between-study heterogeneity variance 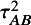 can be defined by the following relationship: 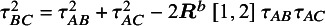, where 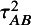 and 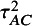 are the variances of random quantities 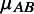 and 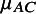 , respectively. These variances are interpreted as the random effects of treatment *B* and *C* relative to a common comparator *A*.

The model parameters described above varied during simulations and were as follows. Two scenarios were considered. The first scenario used a common between-study heterogeneity variance for all treatment comparisons with 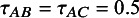 , and the off-diagonal elements of 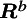 were set to 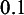 , in which 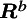 was guaranteed to be positive semi-definite. Despite the assumption of a common between-study heterogeneity variance being widely used in practice, it remains a strong assumption. Thus, we relaxed the assumption of a common between-study heterogeneity variance. Instead, in the second scenario, unequal between-study heterogeneity variances were considered with 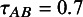 and 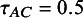 , respectively, and the off-diagonal elements of 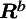 were again set to 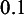 . Under both scenarios, within-study correlations were set at small ( 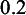 ) and medium ( 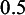 ) magnitudes to explore their impact. Both scenarios reflected a low-to-moderate level of heterogeneity, ranging between 20% and 35% of the total variance; in other words, between-study variance was not large enough to completely dominate within-study variance. We generated closed loops with equal two-arm and three-arm studies into the desired network of studies, with 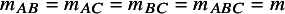 . The true treatment effects for 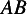 and 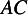 comparisons were mimicked by the IOP data as described in Section 2 and set as 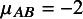 and 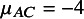 , and 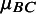 was obtained through the equation: 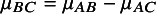 . The simulated study sizes for NMAs were set to 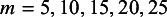 , and 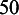 . For each simulation setting, 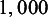 NMA datasets were generated. Using the model parameters described above, continuous data were generated from the multivariate normal distribution in Equation (3). The simulation study was conducted using R software, version 4.2.1.

### 4.2 Simulation results

We evaluated the performance of our proposed method by examining treatment effect estimates, in terms of bias, empirical standard error (ESE), model-based standard error (MBSE), as well as coverage of 95% confidence intervals.

**Figure 2:**
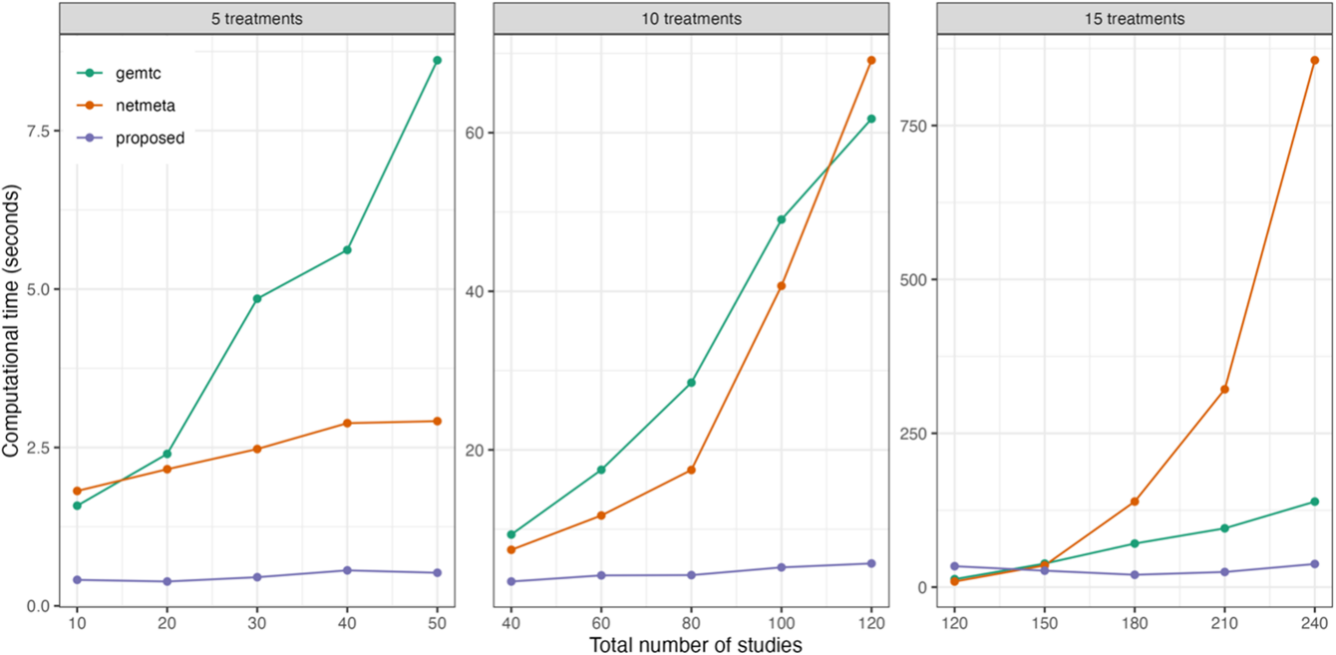
Comparison of computational time for the proposed method and two existing methods implemented in the R packages ‘*gemtc*’ and ‘*netmeta*’, with varying numbers of treatments and studies.

Figure 2 displays the computational time for various NMA approaches. The currently available R packages include ‘*gemtc*’^59^ and ‘*netmeta*’,^60^ in which the ‘*gemtc*’ package employs Bayesian NMA with Markov chain Monte Carlo (MCMC), while ‘*netmeta*’ is designed based on a frequentist random-effects NMA model. We found that initial values could significantly impact the execution time of both ‘*gemtc*’ and the proposed method, whereas ‘*netmeta*’ was less affected by this issue. As the number of treatment comparisons and studies increased, differences in computational time among the three methods became more pronounced. Even though ‘*gemtc*’ and ‘*netmeta*’ required minimal computational time for the scenarios with fewer studies, their computational time increased dramatically as the number of treatment comparisons and studies grew. Conversely, the proposed method generally yielded consistent performance in computational time, irrespective of the number of treatment comparisons or studies. Detailed computational time results are summarized in Table S1 of the Supplementary Material.

The upper panel of Table 1 summarizes simulation results for treatment comparisons 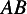 and 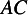 in the scenario with common between-study heterogeneity. Overall, we observed that the proposed composite likelihood-based method yielded approximately unbiased pooled estimates for 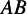 and 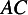 treatment comparisons in most simulation settings. It did not exhibit discernible patterns in 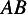 and 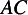 treatment estimates across different magnitudes of within-study correlations (i.e., 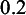 and 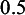 ); in other words, the magnitude of within-study correlations appeared to have a limited impact on the pooled estimates. The confidence intervals computed using the robust sandwich-type variance method demonstrated acceptable coverage probabilities ranging from 88% to 94%, relying on the number of studies. Interestingly, the model-based standard errors appeared somewhat smaller than their empirical standard errors. One possible explanations for this phenomenon is that the proposed method based on composite likelihood provided more efficient inference in large sample settings. However, as widely acknowledged in the literature,^61^ ^–^ ^66^ the variance estimated using the sandwich-type method may be underestimated when the number of studies is below 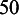 for continuous outcomes.

**Table 1:** Summary of 1,000 simulations with, 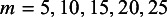 and 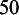 : bias (Bias), empirical standard error (ESE), model-based standard error (MBSE), and coverage probability (CP) of pooled estimates of 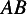 and 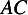 treatment comparisons

Within-study	Number	AB comparison				AC comparison			
correlation	of studies	Bias	ESE	MBSE	CP	Bias	ESE	MBSE	CP
Scenario 1: 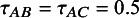 , 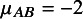 , and 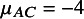									
0.2	5	0.0118	0.3102	0.2860	0.908	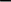 0.0038	0.3261	0.2872	0.900
	10	0.0044	0.2361	0.2074	0.909	0.0037	0.2468	0.2102	0.890
	15	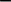 0.0002	0.1925	0.1713	0.905	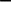 0.0010	0.1932	0.1714	0.914
	20	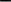 0.0018	0.1646	0.1484	0.911	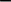 0.0012	0.1647	0.1487	0.922
	25	0.0008	0.1428	0.1333	0.937	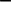 0.0003	0.1456	0.1332	0.920
	50	0.0012	0.1030	0.0945	0.930	0.0025	0.1059	0.0946	0.924
0.5	5	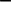 0.0079	0.3456	0.2778	0.876	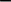 0.0026	0.3284	0.2794	0.883
	10	0.0218	0.2347	0.2010	0.890	0.0130	0.2279	0.2007	0.903
	15	0.0013	0.1817	0.1668	0.923	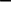 0.0015	0.1948	0.1657	0.896
	20	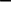 0.0011	0.1594	0.1445	0.921	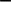 0.0087	0.1572	0.1443	0.925
	25	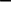 0.0033	0.1386	0.1292	0.939	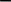 0.0073	0.1410	0.1293	0.926
	50	0.0064	0.1007	0.0917	0.928	0.0031	0.0998	0.0917	0.928
Scenario 2: 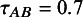 , 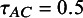 , 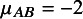 , and 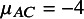									
0.2	5	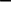 0.0100	0.3473	0.3082	0.892	0.0077	0.3393	0.2967	0.896
	10	0.0108	0.2487	0.2223	0.915	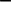 0.0074	0.2396	0.2138	0.911
	15	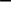 0.0003	0.2032	0.1843	0.925	0.0012	0.1888	0.1762	0.927
	20	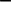 0.0059	0.1744	0.1589	0.930	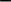 0.0064	0.1691	0.1522	0.918
	25	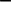 0.0019	0.1565	0.1429	0.917	0.0021	0.1484	0.1363	0.915
	50	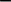 0.0017	0.1146	0.1017	0.919	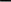 0.0016	0.0996	0.0973	0.942
0.5	5	0.0057	0.3550	0.3022	0.891	0.0066	0.3210	0.2883	0.910
	10	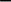 0.0024	0.2454	0.2175	0.910	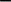 0.0013	0.2265	0.2088	0.923
	15	0.0081	0.2086	0.1786	0.898	0.0134	0.1932	0.1711	0.911
	20	0.0061	0.1786	0.1545	0.904	0.0050	0.1660	0.1480	0.913
	25	0.0001	0.1610	0.1394	0.904	0.0011	0.1447	0.1332	0.911
	50	0.0057	0.1092	0.0987	0.915	0.0076	0.1012	0.0946	0.933

*Note*: Upper panel (Scenario 1): the data-generation mechanism assumes a common between-study heterogeneity variance. Lower panel (Scenario 2): the data-generation mechanism assumes an unequal between-study heterogeneity variance. All results were based on the proposed method without any corrections

**Figure 3:**
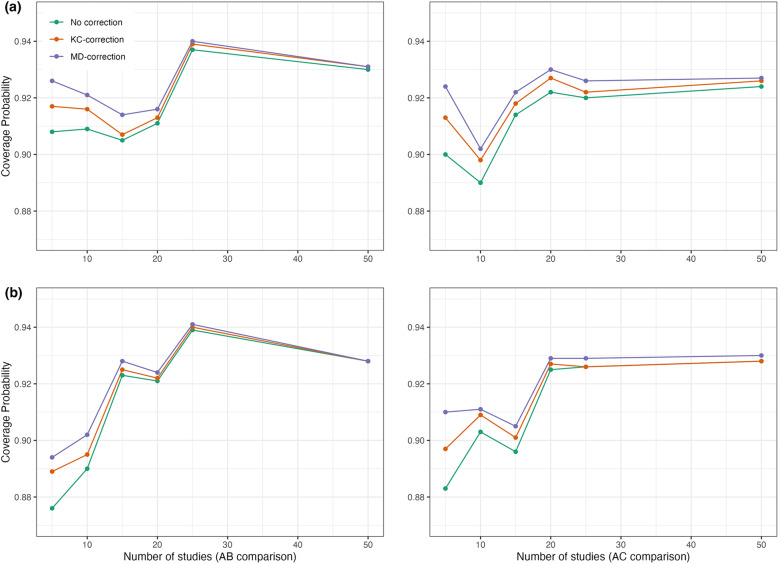
Coverage probabilities of estimated pooled treatment effects for comparisons between treatments 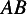 and 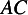 using the proposed method with and without the KC-corrected and MD-corrected sandwich variance estimators under (a) within-study correlation of 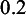 ; and (b) within-study correlation of 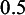 .

To resolve this issue, several alternative bias-corrected sandwich estimators have been proposed to improve a small-sample performance, such as the KC-corrected sandwich estimator^67^ and the MD-corrected sandwich estimator,^63^ among others. As indicated by Li and Redden,^66^ no single bias-corrected sandwich estimator is universally superior; however, a rule of thumb is to choose the KC-corrected method when the coefficient of variation is less than 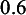 . Through simulation studies, we evaluated whether the coverage probabilities of 95% confidence intervals obtained by the proposed method improved after corrections. Figure 3 displays comparisons of coverage probabilities using the proposed method with and without the KC-corrected and MD-corrected techniques for situations where the number of studies was 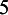 , 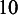 , 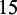 , 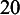 , 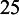 (or even 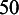 ). We found that the proposed method with corrections exhibited higher coverage probabilities compared to the proposed method without any corrections, particularly when the number of studies was relatively small (e.g., 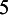 , 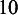 , and 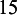 studies). Detailed results with the KC-corrected and MD-corrected methods are provided in Tables S2 and S3 of the Supplementary Material, respectively.

On the other hand, results for the scenario with unequal between-study heterogeneity variance are provided in the lower panel of Table 1. The pooled estimates for 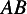 and 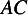 treatment comparisons were generally unbiased. As presented in the lower panel of Table 1, coverage probabilities were acceptable to good across most configurations, yielding coverage probabilities close to the nominal level of 95% (ranging from 89% to 94%). Similarly, variance estimates were adjusted using the KC-corrected and MD-corrected sandwich estimator for smaller studies, as illustrated in Tables S4 and S5 in the Supplementary Material, respectively. As expected, coverage probabilities were slightly improved compared to results obtained from the proposed method without corrections. In summary, simulation results suggested that the proposed method is robust to the magnitude of within-study correlations, regardless of whether the between-study heterogeneity variance is equal or unequal.

## 5 Data application

In this section, we present the results of applying the proposed method to the two published NMAs, primary open-angle glaucoma and chronic prostatitis and chronic pelvic pain syndrome (CP/CPPS), as introduced in Section 2.

### 5.1 Application to primary open-angle glaucoma

The primary outcome of interest, in terms of IOP, was reported in a total of 22,656 patients across 125 studies, evaluating four classes of interventions: 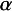 -2 adrenergic agonists, 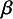 -blockers, carbonic anhydrase inhibitors, and prostaglandin analogs (PAGs). For the IOP outcome, the within-study variances were generally much larger than the between-study variances; the between-study variance was estimated at approximately 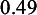 using placebo as a reference. This was reflected by an 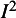 value of 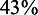 , indicating that the total variation was not completely dominated by the between-study variation. Consequently, within-study correlations might lead to overestimated standard errors of pooled estimates for treatment comparisons if they were not properly accounted for in an NMA. The results of the standard NMA using the Lu and Ades’ approach (shown in the lower triangular matrix) and the proposed method (shown in the upper triangular matrix) without requiring knowledge of within-study correlations are presented in Figure S1 in the Supplementary Material. The results of pairwise meta-analysis are provided in Figure S2 in the Supplementary Material. As displayed in the upper triangular matrix of Figure S1 in the Supplementary Material, all active drugs were likely more effective in lowering IOP at 3 months compared to placebo, with mean differences in 3-month IOP ranging from 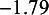 mmHg ( 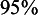 CI, 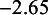 to 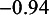 ) to 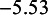 mmHg ( 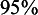 CI, 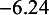 to 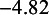 ). Moreover, bimatoprost showed the greatest reduction in 3-month IOP compared to placebo (mean difference = 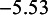 ; 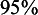 CI, 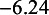 to 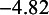 ), followed by travoprost (mean difference = 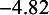 ; 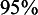 CI, 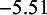 to 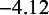 ), latanoprost (mean difference = 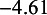 ; 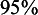 CI, 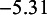 to 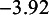 ), levobunolol (mean difference = 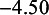 ; 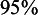 CI, 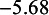 to 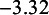 ), taflurpost (mean difference = 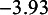 ; 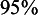 CI, 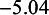 to 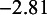 ), and so on. We noted that drugs within the PAG class generally had similar effects on 3-month IOP reduction, except for unoprostone (mean difference = 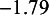 ; 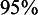 CI, 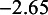 to 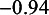 ). Inconsistent results were found in several treatment comparisons when applying the standard NMA and the proposed method, including: brinzolamide versus betaxolol (the standard NMA: mean difference = 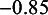 with 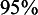 CI, 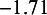 to 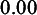 ; the proposed: mean difference = 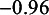 with 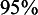 CI, 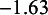 to 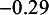 ), carteolol versus apraclonidine (the standard NMA: mean difference = 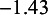 with 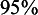 CI, 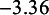 to 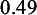 ; the proposed: mean difference = 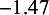 with 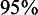 CI, 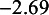 to 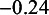 ), tafluprost versus levobetaxolol (the standard NMA: mean difference = 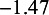 with 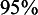 CI, 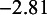 to 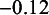 ; the proposed: mean difference = 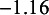 with 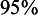 CI, 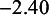 to 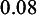 ), brinzolamide versus dorzolamide (the standard NMA: mean difference = 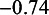 with 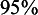 CI, 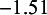 to 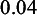 ; the proposed: mean difference = 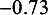 with 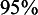 CI, 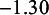 to 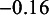 ), and levobunolol versus carteolol (the standard NMA: mean difference = 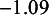 with 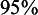 CI, 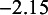 to 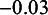 ; the proposed: mean difference = 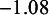 with 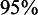 CI, 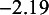 to 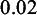 ).

**Figure 4:**
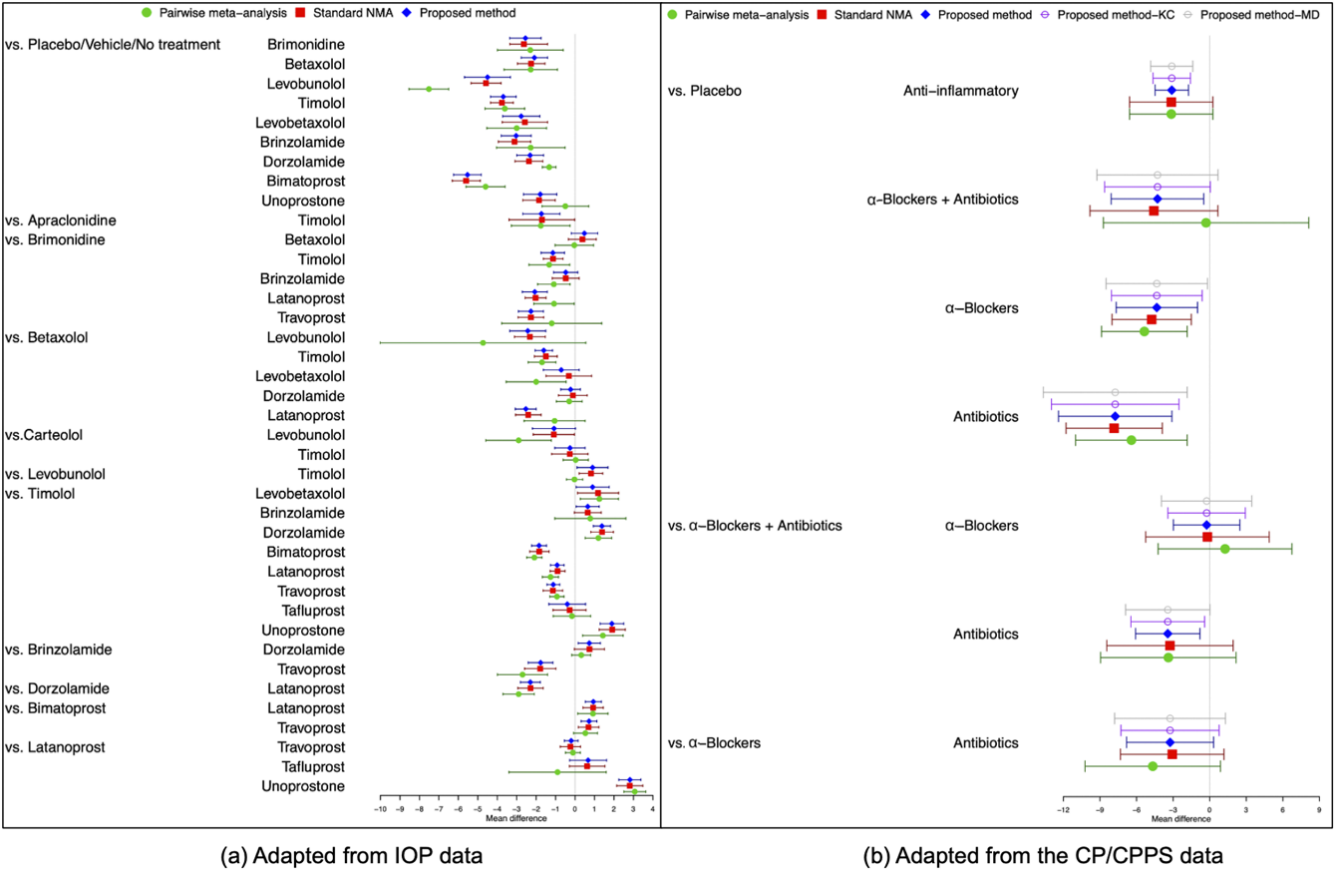
Comparisons of overall relative treatment estimates with 95 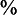 confidence intervals using pairwise meta-analysis approach, the standard NMA based on the Lu and Ades’ approach, the proposed method without any corrections, and the proposed method with KC-corrected or MD-corrected sandwich variance estimators. Each node represents the pooled mean difference for the outcomes of interest.

**Figure 5:**
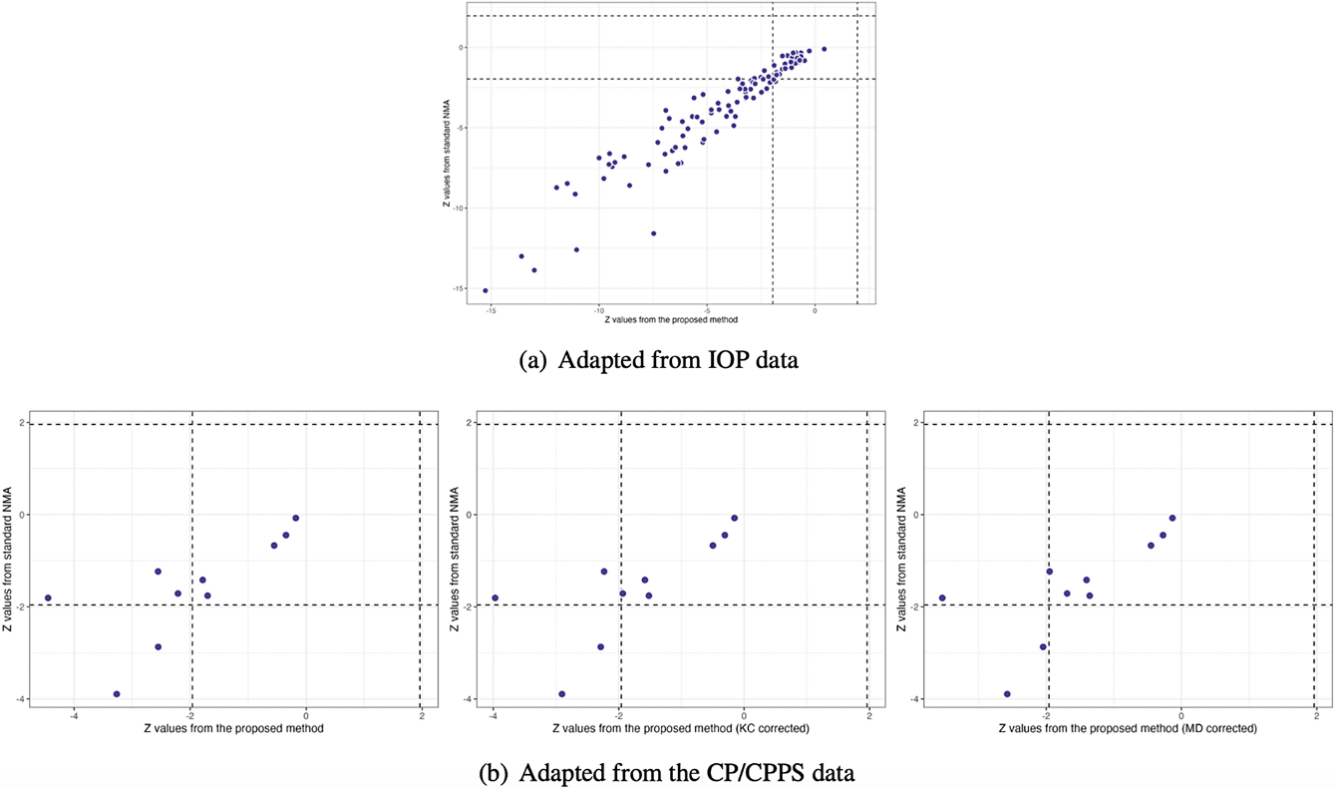
Comparisons of Z values using the standard NMA based on the Lu and Ades’ approach, the proposed method without corrections, and the proposed method with KC-corrected or MD-corrected sandwich variance estimators, respectively

Figure 4(a) illustrates the pooled estimates with corresponding 95% confidence intervals for all treatment comparisons using three approaches: pairwise meta-analysis, the standard NMA based on the Lu and Ades’ approach, and the proposed method. The pairwise meta-analysis provided the direct estimates from all available head-to-head comparisons. As expected, it yielded wider 95% confidence intervals than the other two approaches due to that the indirect evidence was not incorporated into the analysis. The proposed method produced narrower 95% confidence intervals than the standard NMA approach for most treatment comparisons. Figure 5(a) displays a two-dimensional concordance plot of statistical significance, represented by the Z value for both the standard NMA approach and the proposed method, where a Z value less than 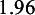 corresponds to a *p*-value greater than 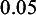 . A few points showed discordant evidence in treatment comparisons between the proposed method and the standard NMA. This discrepancy may be attributed to the fact that the proposed method accounts for non-ignorable effects of within-study correlations, which affect the standard errors of the estimated pooled treatment effects. Figure S3(a) in the Supplementary Material presents the treatment ranking based on the surface under the cumulative ranking curves (SUCRA),^48^ as mentioned in Section 3.3. A higher SUCRA score indicates a superior ranking for 3-month IOP reduction. Consequently, bimatoprost (SUCRA = 98.7%) had the highest SUCRA value for 3-month IOP reduction, followed by travoprost (SUCRA = 66.5%), latanoprost (SUCRA = 51.5%), and levobunolol (SUCRA = 42.5%).

### 5.2 Application to chronic prostatitis and chronic pelvic pain syndrome

In this example, within-study variances are larger than between-study variances for the outcome of NIH-CPSI scores, and thus the effect of within-study correlations cannot be neglected. Due to the limited number of studies, we chose the KC-corrected and MD-corrected methods to adjust the sandwich-type variance estimations, following the rule of coefficient of variation 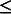 0.6. The NMA results using Lu and Ades’ approach and the proposed method without knowing within-study correlations are presented in Figure S4 in the Supplementary Material. Upon re-analysis, in terms of NIH-CPSI scores, all active drugs showed a statistically significant improvement over placebo, with mean differences of NIH-CPSI scores ranged from 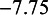 ( 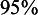 CI, 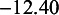 to 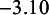 ) to 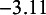 ( 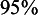 CI, 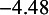 to 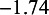 ), as shown in Figure S4 in the Supplementary Material. We found inconsistent results between the proposed method and the standard NMA method. Specifically, 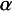 -blockers plus antibiotics (mean difference = 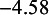 , 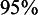 CI: 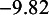 to 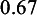 ) and anti-inflammatory agents (mean difference = 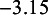 , 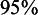 CI: 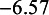 to 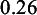 ) did not exhibit significantly greater efficacy than placebo with respect to NIH-CPSI scores when the standard NMA method was applied. Moreover, the proposed method indicated that antibiotics alone were significantly more efficacious than 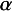 -blockers plus antibiotics (mean difference = 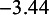 ; 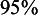 CI, 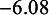 to 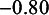 ), a result not observed with the standard NMA method.

Figure 4(b) illustrates the overall relative estimates with 95% confidence intervals for all treatment comparisons using five different approaches, including: pairwise meta-analysis, the standard NMA method, and the proposed method with and without corrections. The results obtained from pairwise meta-analysis yielded wider 95% confidence intervals compared to all other NMA approaches. As expected, the proposed method produced narrower 95% confidence intervals in contrast to the standard NMA using Lu and Ades’ approach. Furthermore, the proposed method without any corrections reported narrower 95% confidence intervals than the standard NMA approach. Corrections for the sandwich-type variance estimations using the KC-corrected and MD-corrected procedures resulted in slightly wider 95% confidence intervals than the proposed method without any corrections for some treatment comparisons. Figure 5(b) displays the concordance plot between the standard NMA approach and the proposed method with or without corrections. Several points showed discordant evidence in treatment comparisons between the proposed method and the standard NMA approach. This discrepancy may arise because the proposed method accounts for the unavailability of within-study correlations, which can affect the standard errors of estimated pooled treatment effects. Figure S3(b) in the Supplementary Material displays the treatment ranking using SUCRA. Overall, antibiotics (SUCRA = 94.6%) were ranked highest for the improvement of NIH-CPSI scores, followed by 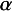 -blockers plus antibiotics (SUCRA = 41.3%) and 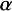 -blockers (SUCRA = 42.4%).

## 6 Discussion

We propose a composite likelihood-based approach to model univariate outcomes in NMAs. The proposed method helps obtain the estimation of overall relative treatment effects in NMAs, even when within-study correlations are unavailable in the original articles. To the best of our knowledge, existing NMA approaches often make assumptions about within-study correlations (assuming them to be known or zero), which can introduce bias if the true within-study correlations are non-negligible. Obtaining within-study correlations typically necessitates a joint analysis of individual participant-level data, often using bootstrapping methods.^68^ ^,^ ^69^ However, this is seldom done unless specific questions about correlations between treatment comparisons are of interest in the included studies. Our proposed method has two key advantages. The first advantage is that the estimation and statistical inference of treatment effects remain valid even if the correlation structure is misspecified. Simulation studies have shown that the proposed method provides nearly unbiased estimates and maintains reasonable coverage rates for 95% confidence intervals across scenarios with common or unequal between-study heterogeneity variances for treatment comparisons. Additionally, as illustrated in two applications, the proposed method is less prone to variance estimation issues than the standard NMA approach when total variation is not dominated by between-study variation. The second advantage of the proposed method is its ability to reduce computational time by circumventing the need to estimate correlation parameters. This improvement is particularly evident when compared to currently available methods for NMAs, such as those implemented in the R packages ‘*gemtc*’ and ‘*netmeta*’ (see Figure 2 and Table S1 in the Supplementary Material).

Nevertheless, there are three potential limitations to the proposed method. First, the proposed method focuses on contrast-based models in NMAs. While NMAs can also be performed using an arm-based approach, there are ongoing debates in the literature regarding the differences between contrast-based and arm-based models.^20^ The arm-based approach offers a promising direction for future NMA modeling. It can potentially alleviate concerns about correlations among contrasts, especially in cases where treatment arm summaries are independent. Under these conditions, the arm-based approach yields the results consistent with the contrast-based method, requiring solely the standard errors of independent treatment summaries along with the inclusion of a fixed study main effect. Piepho and Madden^70^ demonstrated the practical application of an arm-based meta-analysis using the SAS procedures GLIMMIX and BGLMM. Their work highlighted the effectiveness of this approach in circumventing the complexities associated with correlations among contrasts while maintaining concurrent control. However, a key criticism of the arm-based approach is that it might not fully preserve randomization within trials under certain scenarios.^71^ This potential bias in the estimated relative effect is particularly concerning if the assumption of transportable relative treatment effects is violated. It is important to note that when a fixed study main effect is included in the linear predictor, the arm-based NMA utilizes only within-study information, thereby preserving randomization and mitigating this issue^72^ Further research is needed to explore detailed formulations that can optimize the arm-based approach for NMAs. We also encourage researchers to report treatment arm summaries along with their associated standard errors, in addition to reporting contrasts, to provide a more comprehensive understanding of the data.

Second, our simulation studies revealed that the empirical coverage fell below 90% for the 95% nominal level, particularly in studies with limited sample sizes (e.g., 5 or 10 studies). This suggests a potential limitation of the proposed method when dealing with a small number of studies, as the asymptotic properties it relies upon may not fully manifest in such scenarios. Alternatively, the arm-based approach might be less susceptible to this limitation, offering a robust option for analyses involving fewer studies.

Third, because the proposed method is constructed using a composite likelihood-based approach, the variance is estimated through a sandwich-type estimator. This estimator tends to underestimate the true variance, especially when the number of studies is small. This underestimation exacerbates the issue of under-coverage of confidence intervals and inflated type I error rates.^67^ To improve finite-sample variance estimation, we have applied the KC-corrected and MD-corrected sandwich variance estimators^63^ ^,^ ^67^ in our simulation studies and the CP/CPPS application. As a result, the confidence intervals became slightly wider after applying these corrections, leading to improved coverage probabilities compared to the uncorrected method. It would be of interest to investigate how these variations of sandwich variance estimators would improve the coverage probability of the composite likelihood inference in multivariate NMA setting,^73^ and NMA for comparing diagnostic test setting.^74^

In conclusion, this work highlights the importance of considering non-ignorable within-study correlations in network meta-analyses. Ignoring these correlations, particularly when they are non-negligible, can lead to inaccurate standard errors for treatment effect estimates. The proposed composite likelihood-based approach offers an alternative for univariate NMAs when within-study correlation data are not available from original research articles. This method avoids the need for complex individual participant-level data analysis and maintains valid treatment effect estimation, even with misspecified correlation structures. Additionally, it delivers significant computational efficiency gains compared to existing NMA methods.

## Supporting information

Liu et al. supplementary material Liu et al. supplementary material

## Data Availability

The network meta-analysis datasets described in Sections 2 and 5 were obtained from the following published studies: Li et al.,^45^ Wang et al.,^46^ and Thakkinstian et al.^54^ The R code used for data analysis is available at: https://github.com/nbxszby416/uni-CLNMA/tree/main.

## Supplementary material

The supplementary material for this article can be found at https://doi.org/10.1017/rsm.2024.12.
